# Insulinoma: Unraveling the Mystery of a Stealthy Culprit

**DOI:** 10.7759/cureus.74364

**Published:** 2024-11-24

**Authors:** Eram A Mahaldar, Kulsum Khan, Mustafa Khan, Mohammed I Shahbuddin

**Affiliations:** 1 Internal Medicine, Nottingham University Hospitals National Health Service (NHS) Trust, Nottingham, GBR; 2 Endocrinology and Diabetes, Nottingham University Hospitals National Health Service (NHS) Trust, Nottingham, GBR

**Keywords:** 72-hour supervised fast, blood-glucose, minimally invasive pancreatectomy, octreotide scan, pancreatic insulinoma

## Abstract

Insulinoma, a rare pancreatic neuroendocrine tumor, stealthily lurks within the pancreas, often evading detection until its distinctive symptom, recurrent hypoglycemia, comes to the surface. This case report aims to highlight the significance of a multidisciplinary approach in the management of this uncommon neuroendocrine tumor by discussing the diagnostic, therapeutic, and follow-up difficulties encountered in an older adult presenting with atypical symptoms of insulinoma.

## Introduction

Standing at an incidence of approximately four per one million person-years, insulinomas are the most prevalent type of functioning pancreatic neuroendocrine tumor and represent the leading cause of endogenous hyperinsulinemia [1].

Rarely do extra-pancreatic insulinomas occur. These days, metastatic insulinomas are called "aggressive" and non-metastatic insulinomas "indolent." It has been found that patients with indolent insulinomas have a 94% to 100% five-year survival rate, while those with aggressive insulinomas have a 24% to 67% five-year survival rate. The type 1 syndrome of multiple endocrine neoplasia is linked to 5-10% of insulinomas [2].

More than 90% of insulinomas are benign and generally small, well-encapsulated, solitary tumors. Surgical resection is the treatment of choice for insulinomas and the most definitive management [3]. These tumors produce excessive amounts of insulin, leading to frequent episodes of low blood glucose levels. Despite its rarity, prompt diagnosis and management are crucial to prevent potentially life-threatening complications.

When insulinoma occurs, the tumor’s beta cells release insulin autonomously. Insulinoma cells emit insulin continuously, independent of glucose concentration, in contrast to normal beta cells, which adjust insulin secretion in response to blood glucose levels.

Persistent hypoglycemia is caused by this increased insulin secretion, especially when fasting or exercising when glucose availability is restricted [4].

Due to the overwhelming impact of elevated insulin levels, the body's compensatory mechanisms are unable to effectively prevent these occurrences [5].

The uncontrolled insulin secretion from insulinoma persistently drives glucose uptake into the tissues and suppresses hepatic gluconeogenesis. Regardless of food consumption, this prolonged insulin production produces a cycle in which blood glucose levels stay low, especially following fasting or physical activity [6].

## Case presentation

A 73-year-old lady presented with complaints of recurrent hypoglycemia. Her past medical history included Sjogren’s syndrome, arthritis, and immune thrombocytopenic purpura. She had a couple of episodes of falls with dizziness while in the shower over the year before starting to monitor her blood glucose levels. She had never been incontinent or had any episodes of seizures. She had been meticulously monitoring her blood glucose and reviewing her charts and was running low between 3 and 3.5 mmol/L. To combat this, she would eat to keep up with her blood glucose and, as a result, gained significant weight. No history of using any anti-diabetic medication. She would always carry dextrose tablets with her to avoid having low blood glucose and manage with fast-acting carbs.

To investigate, a prolonged supervised fasting test was conducted, and six hours into the fast, a hypoglycemic episode was observed. Her results were as below (Table 1).

**Table 1:** Results following prolonged supervised fasting test

	Patient Result	Reference Range
Blood glucose	2.2 mmol/L	5.6 to 6.9 mmol/L
C-peptide	2,319 ng/ml	0.5 to 2.7 ng/mL
Insulin	35.2 μU/mL	5 and 15 μU/mL

**Figure 1:**
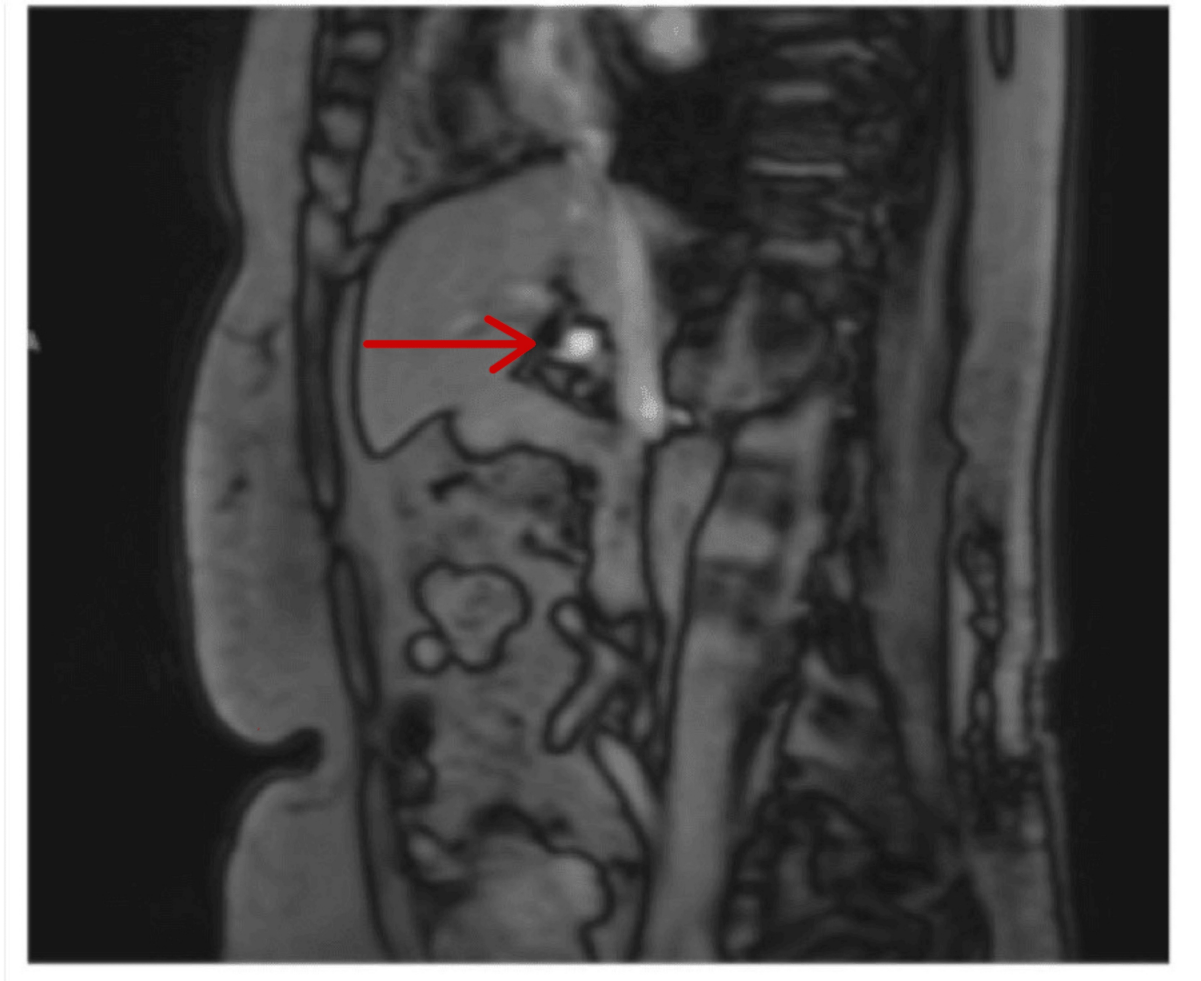
MRI scan showing lesion on pancreatic tail

She had multiple hypoglycemic episodes while in hospital, requiring IV dextrose. An MRI of the pancreas revealed a 19 mm solitary lesion located in the pancreatic tail (Figure 1).

A lesion was seen on cross-sectional imaging, and a biopsy proved it to be a neuroendocrine tumor on EUS sampling. An octreotide scan was done, and to our surprise, no uptake was observed. She was started on diazoxide, which she could not tolerate due to side effects of fluid retention. She was then trialed on octreotide but continued to have recurrent hypoglycemic episodes. 

She was discussed in the Hepatobiliary Multidisciplinary Team meeting, and the outcome was to proceed with surgery as soon as possible as she was struggling with medical therapy.

She underwent a laparoscopic distal pancreatectomy and splenectomy. Postoperatively, she had worsening infection markers, following which a CT of her abdomen and pelvis was done, which showed a small volume of free fluid at the pancreatic surgical bed. She was started on IV antibiotics. She was arranged to have post-splenectomy vaccines at her GP and is to be discharged on lifelong antibiotic prophylaxis.

She was commenced on Humulin I 10 units once daily, but since she did not require it, it was eventually stopped. Her blood glucose level remained below 7.3 mol/L with no episodes of hypoglycemia.

She is regularly followed up on in the outpatient clinic.

## Discussion

Being a diagnostic challenge and, subsequently, a dilemma when it comes to treatment, recent developments have concentrated on increasing diagnostic precision and refining therapeutic strategies. 

Continuous glucose monitoring sensors are being utilized as insulinoma symptoms are sporadic and challenging to record in clinical settings, and this aids in capturing episodic hypoglycemia, which is essential for diagnosis [7].

While the 72-hour fast is still the gold standard for identifying insulinoma, research indicates that a 48-hour fast may be adequate for a large number of patients. According to a study by Hirshberg et al., hypoglycemia was observed in 94.5% of patients with insulinomas within 48 hours of fasting, suggesting that in most cases, prolonging the fast to 72 hours may not be essential [8]. This lessens the strain on patients without sacrificing the sensitivity of the test and also reduces the length of hospital stay. This is especially helpful for people with mild hypoglycemia symptoms and in outpatient settings.

Insulinomas are often small and difficult to find. These can be precisely localized before surgery, with techniques like 68Ga-DOTATATE PET/CT and 18F-DOPA PET scans having demonstrated higher sensitivity in identification when compared to conventional imaging techniques like CT or MRI.

Following the failure of earlier imaging modalities, 68Ga-NOTA-exendin-4 PET/CT was used in one case to localize an insulinoma successfully [9].

Insulinoma localization has been shown to be highly accurate using PET with 18F-DOPA, especially when traditional imaging is unclear. In cases of congenital hyperinsulinism, it is particularly useful for identifying localized lesions [10].

Pharmacological treatment is crucial for individuals who are inoperable, have metastatic illness, or need preoperative stabilization, even if surgical resection is still the only effective treatment.

Peptide receptor radionucleotide therapy (PRRT) is a successful treatment choice for malignant or metastatic insulinomas. Through the modulation of insulin secretion, this medication lowers tumor growth and alleviates hypoglycemia by targeting somatostatin receptors on tumor cells.

In 81% of patients with metastatic insulinomas, PRRT successfully managed hypoglycemia, and 58% of them were able to lessen their need for additional hypoglycemia drugs, according to research published in The Journal of Nuclear Medicine. Following the start of PRRT, the median overall survival was 19.7 months [11].

Diazoxide is a benzothiadiazine derivative used in the medical management of insulinomas, particularly in patients with metastatic disease or who are not candidates for surgery. Its primary mechanism involves inhibiting insulin secretion from pancreatic beta cells, thereby reducing hypoglycemia. Clinical studies have shown its efficacy in controlling hypoglycemia in insulinoma patients, with a 27-year study highlighting its long-term effectiveness and safety. Common side effects include fluid retention, hypertrichosis, gastrointestinal disturbances, and thrombocytopenia, which requires careful monitoring of platelet counts during treatment [12,13]. A study showed that fluid retention was more common in females than males. Five patients displayed unacceptable thrombocytopenia within weeks of diazoxide administration, and the dose was significantly higher in these patients. A careful evaluation of platelet count is also necessary after treatment initiation [13].

Somatostatin analogs like octreotide and lanreotide can also be used to manage hypoglycemia in a sizable percentage of individuals with insulinomas. In a group of 17 individuals, more than half of the hypoglycemic instances were managed with octreotide medication. A positive brief test with subcutaneous octreotide was a superior way to identify responsive patients compared to Octreoscan scintigraphy findings [14].

Everolimus, a mammalian target of rapamycin inhibitor, has shown efficacy in treating pancreatic neuroendocrine tumors, including insulinomas, by inhibiting the mammalian target of rapamycin (mTOR) pathway. This reduces tumor cell proliferation and insulin secretion, alleviating hypoglycemia. A French study found that everolimus therapy normalized blood glucose levels in 11 patients with metastatic insulinomas and refractory hypoglycemia, maintaining the therapeutic effect for a median duration of 6.5 months. However, three patients discontinued everolimus due to cardiac and/or pulmonary adverse events, leading to two deaths [15,16].

Minimally invasive surgical methods, such as laparoscopic and robotic-assisted procedures, have gained popularity for the treatment of insulinomas. These methods are especially beneficial for benign, tiny tumors or those with metastatic disease and are not suitable for surgery.

## Conclusions

Insulinomas are a rare entity and come with a diagnostic dilemma. It can have a significant impact on the quality of life with imminent danger every second of the day. Newer imaging modalities have made it possible to detect these tumors and subsequently have a good treatment outcome.
